# Community partnerships in medical education: Narratives of medical students

**DOI:** 10.1002/puh2.42

**Published:** 2022-11-29

**Authors:** Alexandra M. Cardoso Pinto, Ethan Malone, Nydile Ramesh, Simisola Onanuga, Nikitha Benny, Christine Pettitt, Richard J. Pinder, Shyam Sundar Budhathoki

**Affiliations:** ^1^ Imperial College School of Medicine Imperial College London London UK; ^2^ Department of Primary Care and Public Health School of Public Health Faculty of Medicine Imperial College London London UK

**Keywords:** community engagement, community participation, medical education, partnerships, public involvement, service evaluation

## Abstract

While medical education has traditionally been designed, led and delivered exclusively by clinicians and academics, there has been an increasing shift towards diversifying actors involved in training future generations of health professionals. Public and patient involvement in learning increases the likelihood that learning is relevant to the communities we purport to serve. This article explores the experiences of medical students who were partnered with a community‐based organisation (CBOs) as part of the intercalated Global Health BSc at Imperial College London. Students involved in this programme highlight opportunities to understand the needs of communities they were placed with, beyond what is possible to understand in clinical practice; this is essential to support them in becoming holistic, patient‐centred practitioners. Students also found this opportunity helpful to gain insight into the role and value of the voluntary sector in healthcare and develop transferrable skills in project leadership and management. It is hypothesised that the benefits of this partnership extend to community‐organisations; they gain experience working with students, who provide an external view of their services and may be helpful in the delivery of quality‐improvement projects. Communities could also benefit from interactions with students by sharing insight into their needs and priorities, and in turn, shaping students’ priorities as future health professionals and co‐designers of voluntary‐sector initiatives within the community. Whilst the establishment of these partnerships does not come without its challenges, this article also highlights lessons for students and institutions undergoing similar programmes, including clarification of goals, stakeholder consultation, sustainability of interventions, voicing the community, timetable flexibility and funding.

## INTRODUCTION

Clinicians and academics have historically been the exclusive providers of medical education. Today, provision has diversified to include patients, communities and the general public in the design, delivery and evaluation of medical education [1, 2]. Community involvement with medical education provides a valuable learning opportunity for future healthcare professionals as the voluntary sector becomes increasingly salient in delivering future health and well‐being services. Organisations within the voluntary sector comprise of informed, capable, and generous members of the community who aim to improve community health and wellbeing, often in the absence of financial incentives. Community‐Based Organisations (CBOs) are first‐tier voluntary‐sector organisations which deliver services directly to the community [3]. This paper presents the experiences of Global Health BSc students at Imperial College London, who partnered with CBOs as part of the course's innovative approach to community engagement [4]. By combining these perspectives with wider literature, this article aims to demonstrate the potential role of community organisations in the improvement and delivery of global health education to medical students.

## PERSPECTIVE

This work is based on the perspectives of student‐authors of this piece. Group discussions were held between June and August 2022 to identify learning points and explore the value of partnering with CBOs as part of the Global Health BSc during 2021–2022 academic year. Key quotes by student authors were used to help illustrate each identified theme. Additionally, a literature search was performed on PubMed and Google Scholar between the same dates to identify further examples of community partnerships and their value in medical education. Search domains included “community organisations or voluntary sector,” “partnerships,” and “medical education” with no date or geographical restriction.

### Voluntary‐sector involvement in addressing social inequality

CBOs within the voluntary sector often address the impacts of socioeconomic disparities, including those on health. The COVID‐19 pandemic and resulting "lockdowns" have contributed to worsening mental health; as such, demand for CBOs has increased [5, 6]. CBOs provide a range of services for communities, often complementing the role of policy in addressing the needs of its community [7].

There are also benefits for those who volunteer within these organisations [7]. For example, many volunteers are often from the same community that the CBO caters for. This helps to strengthen support within the local area by providing volunteers with the opportunity to give back to their people. Furthermore, volunteering with these organisations allows individuals to feel a sense of accomplishment, as well as have access to training courses that enhance essential skills, which may lead to future educational and career opportunities [8].

In the United Kingdom (UK), such organisations are a mainstay in some areas of welfare support, financial advice, and activities promoting health, often aimed towards vulnerable groups within society, such as those who are economically vulnerable or elderly people. Such services also complement formally commissioned services and could, in time, replace them. They often work in partnership: for instance, public health initiatives work in collaboration with CBOs to engage hard‐to‐reach groups, as seen with the COVID‐19 vaccine distribution [8, 9].

CBOs play a key role in addressing the Social Determinants of Health (SDH) [7]. These determinants greatly influence an individual's health, as well as the community's well‐being. However, these determinants often cannot be easily addressed through conventional clinical and healthcare means. Literature from the United States highlights how medical education fails to emphasise this important aspect of health, with over 60% of medical students stating that not enough focus is given to the study of SDH [10]. Community placements provide opportunities for students to understand the SDH, their impact on individuals and the power of the voluntary sector to effect change at a community level. This knowledge is transferrable to patient interactions across all specialities and, according to the UK General Medical Council, is essential for doctors to appreciate [11].

### Establishing partnerships between medical schools and the community

Many medical schools have therefore sought to incorporate community involvement into their curricula using various methods with different levels of community participation. Some medical schools, such as the Northern Ontario School of Medicine, have made a “commitment” to integrate community‐engaged and socially accountable medical education into the curriculum which is valued by students [12]. Additionally, Parekh et al. have described a variety of community‐engagement programmes in multiple‐year groups within the Imperial College London medical curriculum, which include working with local schools and community‐engaged quality improvement projects. These initiatives have the potential to support communities, in addressing social and health inequities and establishing greater trust between communities and health professionals [2].

Community engagement programmes have been shown to be beneficial in LMICs, such as Nepal [13]. The B P Koirala Institute of Health Sciences began placing students in rural health settings within Nepal almost 30 years ago, with the aim of improving medical students’ understanding of health needs in rural areas whilst directly supporting the health of these communities [13]. Generally, students are placed outside their teaching hospitals for approximately 1 year, during which they gain a greater understanding of Nepal's health system first‐hand, appreciation for the role of social organisations in community health and hear directly from communities about how services could be improved [13]. Placing students in the community, particularly in underserved settings, has also been described in South Africa, with initiatives dating as far back as 1993 [14]. These initiatives promote problem‐based, experiential learning for healthcare students and encourage interdisciplinary collaboration, whilst providing them with an opportunity to gain insight into community health needs that would otherwise not be appreciated in a traditional medical school environment [14].

An innovative approach to community‐based education, rolled out as Community Group Placement (CGP), has been incorporated into the Global Health BSc at Imperial College London since 2018 [4]. The CGP module comprises a community‐based placement that spans 12 weeks, including classroom preparation, placement‐based activities and report writing [15]. The community placement is an opportunity for mutually beneficial service evaluation and, particularly for students, to contextualise learning from the taught classroom sessions of the course.

### Student experiences in establishing partnerships with CBOs

Student experiences and learning from these placements can be summarised into three themes; the first of which is **learning about the voluntary sector**. Understanding the impact of CBOs enabled students to appreciate their role in health provision and holistic patient care. This placement provides students with the opportunity to familiarise themselves with the services and resources that CBOs offer, and equipped students to be more confident in making use of such resources provided by the community, in addition to clinical resources in their future careers [16].

“So much of health is determined outside healthcare; CBOs help fill the gap that health services fail to meet. They support people beyond giving medication or a one‐hour therapy session. They are there long‐term, a constant source of support and human connection.” (Student A).

This concurs with student feedback from non‐clinical placements at Keele University, which highlighted the importance of non‐clinical community engagement programmes in widening understanding of the role of the voluntary sector in healthcare [17].

In addition, these placements provided students with **opportunities for community engagement**. Meeting service users and witnessing the impact of CBOs on their lives, helped students to understand the needs of the community outside the clinical environment. “*This placement allowed us to gain a deeper understanding of the needs of the community and gave us the confidence to apply this knowledge to improve our future practice*” (Student B). Previous literature has also shown the ability of early patient interaction in increasing healthcare students’ ability to relate to patients’ experiences of illness and the impact of psychological and SDH [18]. It also gave students opportunities to explore barriers faced by community members in receiving support. Gaining these insights may increase empathy and encourage holistic approaches to care in their future practice as healthcare professionals [18, 19]. Moreover, work by the Medical Education Partnership Initiative also describes the value of partnerships between community care providers and medical schools as a means of encouraging health professionals to practice in underserved areas within Sub‐Saharan Africa [20].

Lastly, partnerships with community organisations may support students in **developing as learners**. This placement encouraged students to develop strong management, planning and communication skills, fostered by the partnership between themselves and the CBO. “*We worked collaboratively to establish learning plans and placement goals, and together designed a small‐scale public health initiative which served to help both the local community and the CBO itself*” *(Student C)*. The role of community‐engagement programmes in supporting students to develop communication skills has been described in various community‐engagement initiatives, including the importance of listening to patients [18, 19]. Such collaborations produce doctors who are more apt to successfully partner with individuals and organisations that, whilst not formally part of the health sector, have a substantial health impact, and hence capacity to support patients beyond the clinical experience.

### Wider benefits of successful partnerships

**FIGURE 1:**
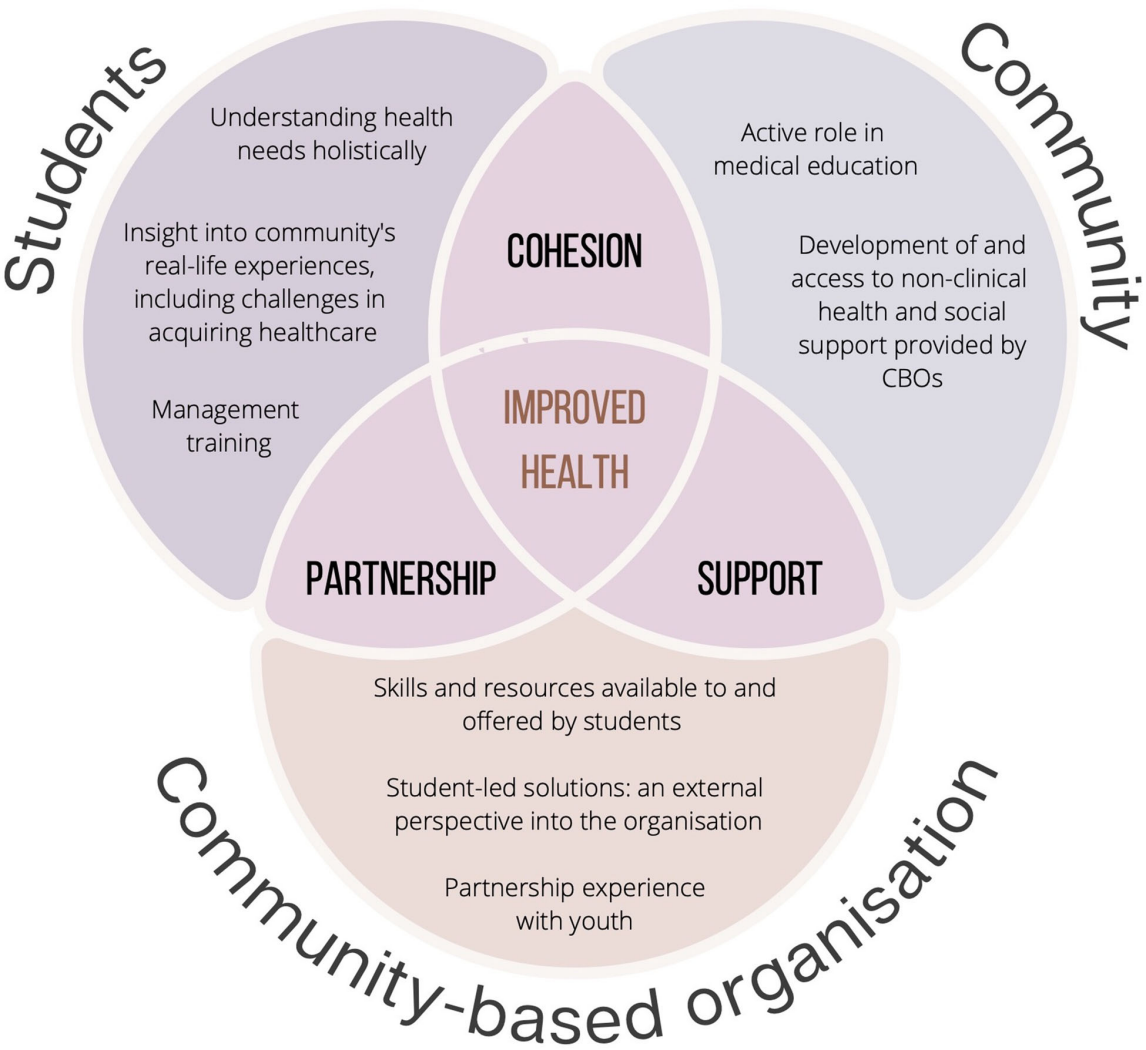
Summary of symbiotic relationship established between students, the community and the community‐based organisations (CBOs) during community placements

The benefits of this placement go beyond those for medical students. An effective placement facilitates the establishment of a symbiotic relationship between students, the community and the organisation (Figure 1). CBOs gain experience partnering with students, receive an external perspective of their services and routes for innovation which may improve future community support. One CBO representative noted specifically that students provided “data expertise,” which enabled increased accuracy when evaluating the impact of their work in their communities [21].

One narrative review of international community engagement initiatives highlighted that community‐based clinicians were appreciative of relationships established with students and universities and the potential of such relationships to improve morale [22]. Some programmes have also resulted in beneficial quality improvement initiatives within host settings, or even the implementation of innovative services [14, 22].

Additionally, community partnerships in medical education empower medical students to play a more active role in the community outside the clinical setting. One such example comes from the Penn State College of Medicine where, following a panel discussion with community leaders involved in a free medical clinic, food pantry and transitional housing programme, some students developed a programme to provide fresh produce to at‐risk patients [16]. A report from Cuba's Latin American Medical School—an institution in which principles of social accountability are strongly embedded into its curriculum—tracked the achievement of its graduates and highlighted examples of how many of them applied skills, from volunteering in disaster‐relief initiatives to setting up a public hospital in a previously underserved community [23]. This emphasises how fostering these values and skills early in medical training may have long‐lasting and far‐reaching impacts.

Finally, community members engaging with students by sharing their perspectives and priorities enables them to lead learning about themselves. This facilitates representative community learning for students and ensures that future healthcare professionals are effectively supported in gaining cultural humility and aligning their priorities with the needs of those they serve.

### Challenges to consider and key lessons for students and institutions

Despite numerous benefits, there are several challenges to establishing successful, sustainable partnerships between students or institutions, and CBOs. These challenges introduce opportunities for exploring future collaborations and strengthening partnerships. For instance, some initiatives have highlighted logistical challenges to placements; strict student timetabling combined with short placements may not align with organisation's availability to receive students, or with the timings of their events [17, 24]. Others highlight financial barriers to establishing additional placements in the community [17, 24, 25]. It is also important to recognise that not all students believe that collaborating with communities is crucial for their medical training or may instead perceive it as a skill to develop later in their careers, which may impact engagement with programmes [16, 25].

Whist student experience was widely positive, it is worth noting that student and organisation priorities do not always align, which poses additional challenges to the collaboration. For instance, students may have pre‐specified academic objectives to achieve, whereas organisations have their own separate aims and performance targets. Ultimately, however, both student and the CBOs aimed to improve community health and well‐being. Thus, this placement enabled reflections on strategies for effective collaboration between students and CBOs, to maximise benefits for all parties.

Key messages for success for any student undergoing a community placement with CBOs and their institutions include:

- **Establishing goals**: students and CBOs should clarify goals early on, to ensure that all aims will be achieved during the placement.
- **Involving stakeholders**: communicate with all stakeholders and learn how they cooperate to meet the needs of local people. This can be done by attending events and meetings which will help students gain valuable insight into the organisation and its community.
- **Sustainable interventions**: throughout the placement, students should develop an intervention that meets the needs of local people in a way that is achievable for the CBO and brings long‐lasting change to the local community. Asset‐based approaches may facilitate this, especially if proposed in small, incremental steps.
- **Voicing the community**: students should ensure that the local community is kept at the heart of all decision‐making and recognise that as the community is a stakeholder itself, ideal improvements are likely to be voiced by community members themselves.
- **Flexibility in timetabling**: enabling students and organisations to arrange visits at different days and times during the week, or facilitating long‐term placements, to ensure students participate in a variety of activities led by the organisation.
- **Funding**: supporting the organisation with costs incurred by visiting students, and ensuring feasible travel distances, or financial support for travel or accommodation costs for students.

## CONCLUSION

CBO‐partnered community placements enable the establishment of a mutually beneficial relationship between medical students, CBOs and the community they serve. Therefore, we invite educators and institutions to consider implementing CBO‐partnered learning approaches and placements into medical curricula globally. These placements have the potential to shape medical student understanding of the role these organisations play in population health and bring them closer to people, supporting them to become empathetic professionals, better equipped to support future patients, communities, and society.

## AUTHOR CONTRIBUTIONS

Alexandra M. Cardoso Pinto was responsible for conceptualisation and overall leadership of the work. Shyam Sundar Budhathoki supervised the work. All authors contributed to the writing and revision of the manuscript.

Ethan Malone: Writing – original draft; Writing – review & editing. Nydile Ramesh: Writing – original draft; Writing – review & editing. Simisola Onanuga: Visualization; Writing – original draft; Writing – review & editing. Nikitha Benny: Writing – original draft; Writing – review & editing. Christine Pettitt: Writing – original draft; Writing – review & editing. Shyam Sundar Budhathoki: Supervision; Writing – review & editing

## CONFLICTS OF INTEREST

The manuscript is authored by students who completed the Community Group Placement module as part of the Global Health BSc at Imperial College London and course leads who designed and implemented the module. Shyam Sundar Budhathoki is an Editorial Board member of Public Health Challenges, and a co‐author of this article. To minimize bias, they were excluded from all editorial decision‐making related to the acceptance of this article for publication.

## Data Availability

Data sharing not applicable.
